# Responses of soil nitrogen fixation to *Spartina alterniflora* invasion and nitrogen addition in a Chinese salt marsh

**DOI:** 10.1038/srep20384

**Published:** 2016-02-12

**Authors:** Jingxin Huang, Xiao Xu, Min Wang, Ming Nie, Shiyun Qiu, Qing Wang, Zhexue Quan, Ming Xiao, Bo Li

**Affiliations:** 1 Ministry of Education Key Laboratory for Biodiversity Science and Ecological Engineering, Fudan University, 2005 Songhu Rd, Shanghai 200438, Peoples R China; 2 Shanghai Academy of Environmental Sciences, 508 Qinzhou Rd, Shanghai 200233, Peoples R China; 3 Department of Microbiology and Microbial Engineering, School of Life Sciences, Fudan University, 2005 Songhu Rd, Shanghai 200438, Peoples R China; 4 Department of Biology, Shanghai Normal University, 100 Guilin Rd, Shanghai 200234, Peoples R China

## Abstract

Biological nitrogen fixation (BNF) is the major natural process of nitrogen (N) input to ecosystems. To understand how plant invasion and N enrichment affect BNF, we compared soil N-fixation rates and N-fixing microbes (NFM) of an invasive *Spartina alterniflora* community and a native *Phragmites australis* community in the Yangtze River estuary, with and without N addition. Our results indicated that plant invasion relative to N enrichment had a greater influence on BNF. At each N level, the *S. alterniflora* community had a higher soil N-fixation rate but a lower diversity of the *nifH* gene in comparison with the native community. The *S. alterniflora* community with N addition had the highest soil N-fixation rate and the *nifH* gene abundance across all treatments. Our results suggest that *S. alterniflora* invasion can increase soil N fixation in the high N-loading estuarine ecosystem, and thus may further mediate soil N availability.

Biological nitrogen fixation (BNF) is a major part of N cycling. The total terrestrial N-fixation rate is about 58 Tg N yr^−1^ 1, accounting for more than 97% of new N input to unmanaged terrestrial ecosystems^2^,^3^. BNF rates vary among ecosystems^4^. Many biotic and abiotic factors such as N-fixing microbe (NFM) diversity, soil C:N ratio, and soil P level can affect BNF^5^,^6^. Recently, some studies have found that plant invasion and N enrichment can considerably affect BNF. For example, *Ageratina adenophora* invasion significantly increases the diversity and abundance of N-fixing bacteria in a forest ecosystem^7^. The amount of fixed N by invasive *Myrica faya* (18 kg N ha^−1^ yr^−1^) is far greater than by a native plant (0.2 kg N ha^−1^ yr^−1^)^8^. In addition, N enrichment is shown to change N-fixing bacterial community and abundance of functional N-fixation genes^9^,^10^. Although these studies suggest that plant invasion and N enrichment can influence BNF, their combined effects remain unclear.

Plant invasions are major threats to native ecosystems. Invasive plants can destroy native habitats, replace native plants, and change nutrient cycling and soil properties^11^,^12^. In the eastern coast of China, exotic grass *Spartina alterniflora* was introduced in 1979^13^. After its initial appearance on the Chongming Island located in the Yangtze River estuary in mid-1990′s, it has replaced native *Phragmites australis* and *Scirpus mariqueter* and becomes a dominant plant species there^11^. *S. alterniflora* invasion changes soil microorganism community^14^,^15^ and stimulates greenhouse gas fluxes^16^. *S. alterniflora* invasion also increases soil C and N pool sizes^17^. Our previous study suggests that N concentration of *S. alterniflora* litter increases as it decomposes in the air and soil, whereas N concentration of *P. australis* litter decreases^18^.

In addition, the Yangtze River estuary has long suffered from high N loading due to human activities. It is estimated that approximately 1.339 × 10^6^ t of dissolved inorganic N (DIN) has been discharged to the Yangtze River estuary in 2007^19^. N enrichment in the Yangtze River has increased from 13.6 Tg in 1990 to 19.8 Tg in 2000, and the proportion of anthropogenic N increased from 48% in 1980 to 68% in 2000^20^. DIN concentrations are predicted to be 2.2–3.0 mg L^−1^ between 2020 and 2050^19^. N enrichment can lead to altered plant composition^21^, promote biological invasion^22^,^23^, and alter C/N cycling^24^,^25^ and microbial activity^26^. In the Yangtze River estuary, for example, N enrichment stimulates CH_4_ emission in the *S. alterniflora* community but does not affect the emission in the native plant communities^27^. In addition, N enrichment stimulates N_2_O emissions in both invasive *S. alterniflora* and native *P. australis* communities^28^.

Here we conducted an experimental study in the Yangtze River estuary. Because of the positive relationship between NFM and plant C input to soil^29^, we hypothesized that invasive *S. alterniflora* relative to native *P. australis* has a higher simulative effect on soil N fixation. Previous studies suggest that N enrichment can affect soil N-fixation^2^. Therefore, we further hypothesized that N enrichment can regulate soil N fixation after plant invasion.

## Materials and Methods

### Experimental design

**Figure 1:**
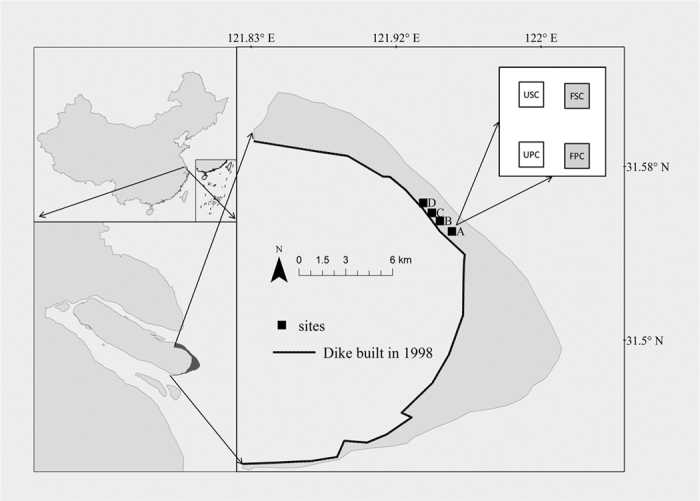
Sketch map of our study site in the Chongming Island of the Yangtze River estuary (31°25′–31°38′N, 121°50′–122°05′E). This figure was generated using ArcGIS 10.2 (ESRI, Redlands, CA, USA). UPC: unfertilized *Phragmites australis* community; USC: unfertilized *Spartina alterniflora* community; FPC: fertilized *Phragmites australis* community; FSC: fertilized *Spartina alterniflora* community.

We conducted our study in the Dongtan National Nature Reserve, located in the Yangtze River estuary of China (31°25′–31°38′N, 121°50′–122°05′E) (Fig. 1). The area has an elevation of 2–6 m, and receives sediment and nutrients from the tide^30^. The area has a subtropical monsoon climate and receives most of its rainfall between May and September. The growing season begins in April and ends in October^31^. We established 4 plots which had approximately 500 meters far from each other (Fig. 1). All plots was at the same elevation with similar sediment textures and received the same tidal timing. Each plot had a factorial combination of two plant communities and two N levels (UPC: unfertilized *Phragmites australis* community; USC: unfertilized *Spartina alterniflora* community; FPC: fertilized *Phragmites australis* community; FSC: fertilized *Spartina alterniflora* community). All plant communities grew naturally in this study. The size of each plot was 0.8 × 0.8 m. In each plot, the same type of plant communities were approximately 2 meters apart. The distance between communities of different plant types was no more than 10 meters. To simulate N enrichment, urea (100 g N m^−2^ y^−1^) was added to the fertilized communities during growing season (April to August) in 2012 and 2013. To eliminate possible litter effects, litter was removed thoroughly at the beginning of this study.

At the end of the experiment, aboveground biomass was harvested, oven dried and weighted. Five soil cores were collected from the top 15 cm soil of each plot by using a 5-cm-diameter auger. Collected rhizosphere soils were within the densely rooted portion of the soil profile. After being mixed thoroughly, soil samples were hand-sifted to remove residual roots/rocks and separated into two parts. One part was stored at −80 °C for analyzing N-fixing microbes, and the other part was stored at 4 °C for measuring N-fixation rate and soil properties.

### Measurements of N-fixation rate and soil properties

N-fixation rate was determined by a ^15^N_2_-based method as described by Hsu and Buckley (2009). Briefly, duplicate subsamples (5 g each) from each soil sample were weighted into two tubes. The headspace of one tube was replaced with 20% oxygen and 80% ^15^N_2_ mixed gas, while the headspace of another tube was replaced with 20% oxygen and 80% ^14^N_2_ mixed gas. Then, these tubes were incubated at 25 °C for 9 days in the dark. The quantities of ^15^N and ^14^N in the incubated soils were determined by an isotope ratio mass spectrometer (EA Flash2000-Delta Advantage, Thermo Electron Corporation, USA). The potential N-fixation rate was calculated by subtracting the quantity of ^15^N in labeling tube from the quantity of ^15^N in the control tube^32^.

Soil pH was determined *in situ* using an IQ150 pH Meter (Hach, USA). Because urea can naturally change to ammonia within a few days^33^, we did not measure urea for calculating soil N availability. NH_4_^+^ and NO_3_^−^ were extracted from fresh soil samples using 2 M KCl (1:4, w/v) and assayed colorimetrically with a discrete auto-analyzer (SmartChem200, WestCo, America). A soil suspension was prepared using 3 g of air-dried soil and 40 ml deionized water, and then was analyzed with a Laser particle size analyzer (OMEC LS-CWM(3), China) to determine soil particle size. Soil salinity (1:5, w/v) was determined using a conductivity meter (Metler SevenEasy, Switzerland). Soil orthophosphate (PO_4_^3−^) was extracted by using 0.5 M NaHCO_3_ (1:20, w/v) and was assayed with a discrete auto analyzer (SmartChem200, WestCo, America). Soil total N (TN) and total carbon (TC) contents were determined with a C/N Soil Analyzer (Flash EA 1112 Series, Thermo, Italy).

### NFM community

Total soil DNA (0.5 g soil) was extracted using a FastDNA^®^ SPIN Kit for Soil (MP Biomedicals, US) following the manufacturer’s instructions. The number of NFM was quantified by a SYBR Green I-based qPCR method. The *nifH* gene primers (PolF: TGC GAY CCS AAR GCB GAC TC; PolR: ATS GC CAT CAT YTC RCC GGA) were used in this study^34^. Each reaction tube contained 0.5 μl of template DNA (5–10 ng), 10 μl of SYBR^®^ Premix Ex Taq™ (Takara, China), 0.5 μl (10 μM) of each primer, 0.5 μl of BSA (1 μg ul^−1^), 0.4 μl of ROX reference dye (50X), and 7.8 μl of sterile distilled water. A known copy number of plasmid DNA carrying the *nifH* gene was used as a template to create a standard curve. Quantitative PCR was carried out using an ABI7900 (ABI, USA). Thermal cycling was conducted using the following steps: 95 °C for 3 min, followed by 4 cycles of touchdown PCR (95 °C for 15 s, 63 °C for 25 s, and 72 °C for 45 s, with a temperature decrease of 2 degrees every two cycles), then 40 cycles of 95 °C for 15 s, 55 °C for 25 s, and 72 °C for 45 s. The results were analyzed using ABI SDS software (version 2.4, ABI, USA).

The template DNAs isolated from the subsamples of each plot were pooled so that each subsample was equally represented. The *nifH* gene diversity and NFM community were characterized by 454-pyrosequencing. The *nifH* gene primers IGK3 (GCI WTH TAY GGI AAR GGI GGI ATH GGI AA) and DVV (ATI GCR AAI CCI CCR CAI ACI ACR TC) were used. Before PCR, the primers were barcode labeled. PCR amplification was performed with an initial denaturation at 94 °C for 10 min, then 30 cycles of 94 °C for 30 s, 58 °C for 30 s and 72 °C for 30 s, followed by a final extension at 72 °C for 10 min^35^. The PCR products were purified and then used for 454-pyrosequencing. Approximately 10,000 DNA sequences were obtained from each sample. Next, the *nifH* gene DNA sequences were filtered using quality files, and the remaining sequences were trimmed with barcodes and forward primers. These sequences were converted to amino acid sequences using the Framebot tool in the RDP function gene pipeline (http://fungene.cme.msu.edu/FunGenePipeline/). The sequences that did not correspond to the *nifH* gene or that had a termination codon in the middle were removed. The sequences that were too long, too short, or had ambiguous bases were also removed. The remaining quality sequences were aligned with the *nifH* gene database^36^, and poorly aligned sequences and chimeras were removed. Then, the remaining clean sequences were used for OTU-based analysis. OTU was defined using 90% and 80% sequence-identity cutoffs. Richness (ACE and Chao1) and diversity analyses were performed after the sequence number in each sample had been normalized. All steps of the quality sequence analysis and diversity analysis were performed using Mothur software, version 1.8^37^. Normalized *nifH* OTUs at a sequence similarity cutoff of 80% were also used for Jackknife clustering analysis which was carried out using the online free UniFrac software (http://unifrac.colorado.edu). The unifrac metric through a weighted Jackknife clusters method was determined by relative sequence abundances and genetic distances of the four treatments^38^. The representing sequences of the OTUs (>1% in the total sequences) in combination with the reference sequences from other two related studies^39^,^40^ were used to construct a phylogenetic tree. The accession numbers are shown in the phylogenetic tree. All of the clean sequences were identified using Blastx (http://blast.ncbi.nlm.nih.gov/) to determine functional compositions.

### Data analyses

Visual maps for the location of the Chongming Island was created using ArcGIS version 10.2 (ESRI Inc., Redlands, CA, USA). Copy number of the *nifH* gene per gram of dry soil was log converted [log (the copy number of *nifH* g^−1^ dried soil]. We used two-way ANOVA to analyze the effects of plant type and N enrichment on N-fixation rate and soil properties. Canonical correspondence analysis (CCA) was used to examine the relationships between OTU compositions and environmental factors^41^. All statistical analyses were performed with R software 3.1.2 (R Core Team, 2014).

### Nucleotide Sequence Accession Numbers

The partial *nifH* sequences recovered in this study were available in the NCBI GenBank Short Read Archive (SRA) under Accession No. SRP066209.

## Results

**Figure 2:**
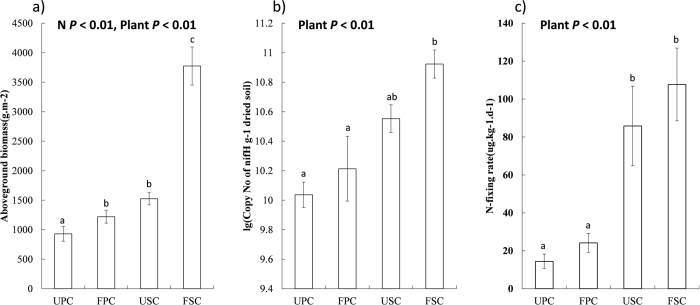
Aboveground biomass (**a**), copy number of the *nifH* gene (**b**), and nitrogen fixation rate (**c**). UPC: unfertilized *Phragmites australis* community; USC: unfertilized *Spartina alterniflora* community; FPC: fertilized *Phragmites australis* community; FSC: fertilized *Spartina alterniflora* community. Treatments followed by the same letters are not significantly different.

*S. alterniflora* biomass was significantly higher than *P. australis* biomass at the same N level (Fig. 2a). N enrichment significantly stimulated biomass of both native and exotic plants (Fig. 2a). Plant type had a significant effect on the copy number of *nifH* gene (*P* < 0.01). The highest copy number of *nifH* gene was found in FSC (Fig. 2b). Plant type also significantly affected N-fixation rate (*P* < 0.01). N-fixation rate was significantly higher in the soils of *S. alterniflora* communities than in those of *P. australis* communities (Fig. 2c). In addition, the copy number of the *nifH* gene was positively correlated with N-fixation rate (*R*^2^ = 0.95, *P* = 0.03).

**Figure 3:**
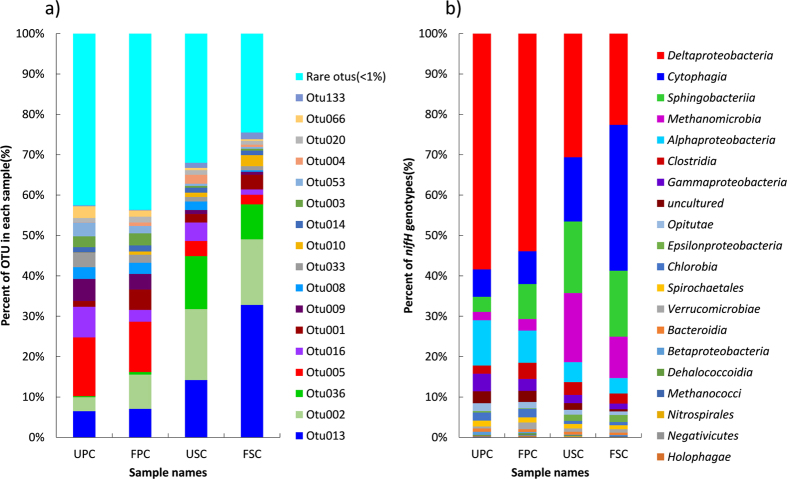
Proportions of *nifH* gene types at 80% similar cutoff (**a**) and N-fixation bacterial types (**b**). UPC: unfertilized *Phragmites australis* community; USC: unfertilized *Spartina alterniflora* community; FPC: fertilized *Phragmites australis* community; FSC: fertilized *Spartina alterniflora* community.

**Figure 4:**
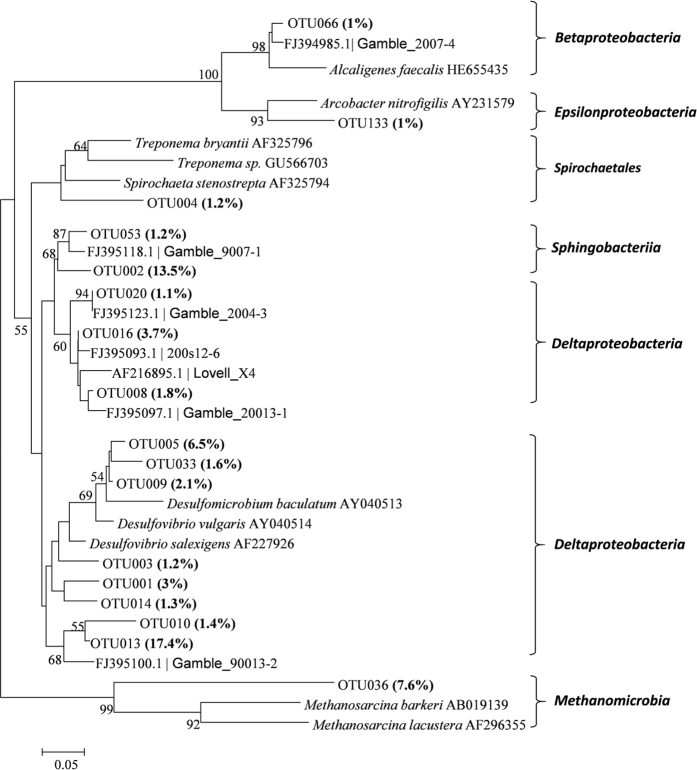
Phylogenetic tree based on the representative sequences (>1% abundance in the total sequences) of the *nifH* gene. The percentages in parentheses indicate those in the total number of sequences. The sequences which are named with Gamble and Lovell are the sequences from Gamble’s^39^ and Lovell’s^40^ studies, respectively. Bootstrap values (%) are only shown when they are greater than 50%.

**Figure 5:**
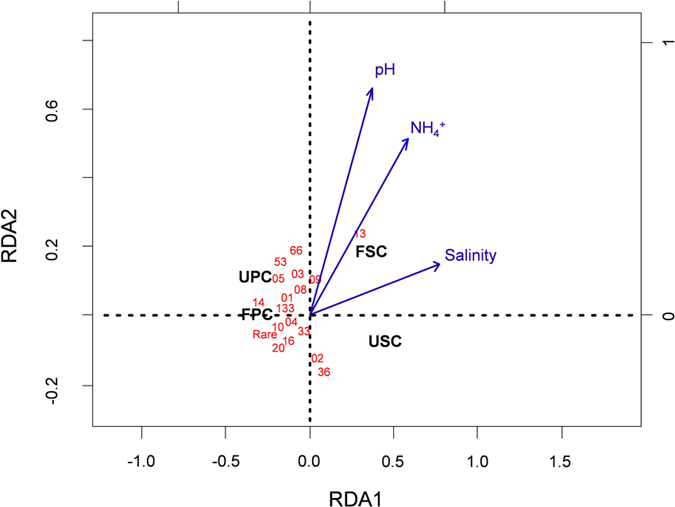
Result of CCA tri-plot. The direction of an arrow indicates the steepest increase in the variable, and the length indicates the strength relative to other variables. UPC: unfertilized *Phragmites australis* community; USC: unfertilized *Spartina alterniflora* community; FPC: fertilized *Phragmites australis* community; FSC: fertilized *Spartina alterniflora* community. The numbers are OTU names (e.g. 36 is OTU036).

The OTU number and richness of the *nifH* gene were higher in the soils of *P. australis* communities compared with the *S. alterniflora* communities (Table 1). N enrichment increased the chao1 and ACE indices in each type of plant community (Table 1). Soil *nifH* gene diversity was generally higher in the *P. australis* communities than *S. alterniflora* communities (Table 1). Compared with the *P. australis* communities, the soil of *S. alterniflora* communities had higher proportions of OTU013, OTU002, and OTU036, and lower proportions of OTU005, OTU009, OTU008, OTU003, and OTU014 while N enrichment had little effect on the composition of the *nifH* gene (Fig. 3a). All dominant OTUs in this study matched the known N-fixation microbial orders (Fig. 4). Generally, the most dominant OTUs accounting for 41.1% of the total sequences were associated with the class *Deltaproteobacteria*. OTU013, OTU020, and OTU002 were similar to the sequences discovered in a previous study^39^,^40^ (Fig. 4). Based on the CCA soil pH had the strongest effect on soil NFM compositions (Fig. 5). OTU013 positively correlated with pH, NH_4_^+^, and salinity (Fig. 5). But OTU002, OTU020, OTU016, OTU033, OTU004 negatively correlated with these environmental factors (Fig. 5).

**Figure 6:**
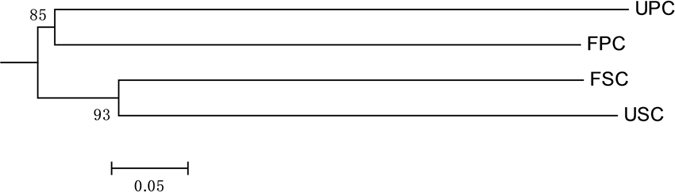
Jackknife clustering analysis of *nifH* gene compositions. The unifrac metric through a weighted Jackknife clustering method was determined by relative sequence abundances and genetic distances of the four samples, and all of the calculation process were carried out using the online free UniFrac software (http://unifrac.colorado.edu)^40^. The numbers (85 and 93) are Jackknife resampling percentages. UPC: unfertilized *Phragmites australis* community; USC: unfertilized *Spartina alterniflora* community; FPC: fertilized *Phragmites australis* community; FSC: fertilized *Spartina alterniflora* community.

The soils of *S. alterniflora* communities had lower proportions of *Deltaproteobacteria*, *Alphaproteobacteria* and *Gammaproteobacteria* but higher proportions of *Cytophagia*, *Spirochaetales*, and *Methanomicrobia* in comparison with the soils of *P. australis* communities (Fig. 3b). Jackknife cluster analysis suggests that *S. alterniflora* communities had similar *nifH* composition for two N treatments as clustered into one clade. However, the *nifH* compositions in the *P. australis* communities were clustered into different clades for two N treatments (Fig. 6).

There were no significant differences in the salinity, NO_3_^−^/NH_4_^+^ concentrations, and soil particle size across all treatments. However, the soils from the *S. alterniflora* communities had higher C/N concentrations but lower P concentration in comparison with the soils from the *P. australis* communities, leading to higher ratios of soil C:P and N:P in the *S. alterniflora* communities (Table 2).

## Discussion

It is well known that soil microbial diversity and activity are mediated by environmental properties. Plant invasions are shown to change belowground diversity and processes^42^,^43^. Our findings add to a growing body of evidence that plant invasions can increase soil N-fixation rate^8^,^12^,^44^, possibly because invasive plants has higher stimulative effects on their rhizosphere bacterial activity in comparison with co-occurring native plants. Many studies suggest that soil microbial activity is highly correlated with plant biomass production and C input to soil. In a saltmarsh meadow, for example, soil N fixation rate increases with increasing plant primary productivity^29^. In the ecosystem we studied, the *S. alterniflora* community had much higher aboveground biomass (Fig. 2a), litter production, soil organic matter, microbial biomass, and soil respiration rate than the native *P. australis* community^16^,^45^. Therefore, increased soil C availability due to *S. alterniflora* invasion might be responsible for the high NFM number and N fixation rate.

On the other hand, soil nutrient availability has positive effects on NFM community and N-fixation rate^2^. *S. alterniflora* invasion increases soil N pool size and availability of the Yangtze River estuary because of the invasion-induced increase in soil N intercept through sedimentation^18^,^30^. In addition, litter of the invasive plant can decompose more rapidly than that of the native plant, leading to a faster nutrient recycling rate of the invasive plant^18^,^30^. As a result, a high availability of soil nutrient after *S. alterniflora* invasion may lead to a stimulation of BNF.

We found that *S. alterniflora* invasion changed soil N:P ratios (Table 2). These changes in soil stoichiometric properties might also affect NFM community. In P-limited (relative to N) ecosystems, for example, N fixation rate is negatively correlated with N:P ratio in grassland and tropical forest soils^46^,^47^. However, the soils of *S. alterniflora* communities relative to *P. australis* communities had higher N:P ratios as well as higher N fixation rates (Fig. 2b, Table 2). These results suggest that soil nutrient factors affecting N fixation rate are different between ecosystems^2^. Although soil N concentration is relatively higher in the Yangtze River estuary compared with other nutrient-limited ecosystems^46^,^47^, N enrichment largely stimulated plant growth (Fig. 2a), possibly suggesting that N relative to P may play a more important role in plant growth in Yangtze River estuary.

Our results are consistent with other studies conducted in salt marshes 39,40, showing that the most dominant NFMs belong to *Deltaproteobacteria* (Figs 4 and 5). Our results indicated that *S. alterniflora* invasion had larger effects than N enrichment on the N-fixation rate and NFM community (Fig. 2c). Soil N fixation can be directly stimulated by increased plant production. However, the main way that soil N fixation is affected by N enrichment is believed to be indirect plant stimulation^48^. In addition, we found that two-year N enrichment had little effect on soil concentrations of TN, NH_4_^+^, and NO_3_^−^ (Table 2), possibly because litter was removed in our study, it would reduce litter N retention and N input. In addition, perhaps because the ecosystems in the Yangtze river estuary are so eutrophic that N addition is buffered. This may also lead to a greater influence of plant invasion on N-fixation rate and NFM community than that of N enrichment.

Overall, our research demonstrated that *S. alterniflora* invasion changed the functional microbial community for fixing N and increased N-fixation rate in an invaded estuarine ecosystem. However, N enrichment had little effect on this functional process compared with plant invasion. Our study would improve our understanding of ecosystem consequences caused by plant invasion in the highly disturbed estuarine ecosystem. Our results also suggest that there could be a positive feedback between *S. alterniflora* invasion and eutrophication. The *S. alterniflora* invasion plus N addition may increase N fixation and N input, further accelerating the invasion and N pollution. Thus, the spread of *S. alterniflora* may aggravate the eutrophication in China’s coastal zones. Our results are potentially useful for the modeling, prediction, and management of plant invasion under eutrophication in coastal wetland ecosystems.

## Additional Information

**How to cite this article**: Huang, J. *et al.* Responses of soil nitrogen fixation to *Spartina alterniflora* invasion and nitrogen addition in a Chinese salt marsh. *Sci. Rep.* **6**, 20384; doi: 10.1038/srep20384 (2016).

## Figures and Tables

**Table 1:** Diversity of bacterial *nifH* gene.

Similarity cutoff			90%	
Sample names	No of sequences	OTUs	Chao1	ACE
UPC	1450	473	1047.135	1696.042
FPC	1509	560	1106.391	2188.452
USC	1309	284	460.9531	680.6599
FSC	1274	207	484.8889	633.2822
**Similarity cutoff**			**80%**	
UPC	1450	260	455.7347	655.5646
FPC	1481	319	547.7639	1019.613
USC	1388	183	249.9783	279.7492
FSC	1356	143	275.1429	337.6031

UPC: unfertilized *Phragmites australis* community; USC: unfertilized *Spartina alterniflora* community; FPC: fertilized *Phragmites australis* community; FSC: fertilized *Spartina alterniflora* community.

**Table 2:** Soil properties with ANOVA’s summary for the different treatments.

Plant type	N enrichment	Salinity (g L^−1^)	PH	NH_4_ (mg L^−1^)	NO_3_ (mg L^−1^)	Total N (g kg^−1^)	Total C (g kg^−1^)
*Phragmites*	No	2.42 ± 0.49a	7.5 ± 0.11a	7.93 ± 0.72a	0.32 ± 0.09a	1.29 ± 0.3a	20.1 ± 1.03a
*Phragmites*	Yes	2.55 ± 0.5a	7.38 ± 0.2a	7.04 ± 0.4a	0.85 ± 1.09a	1.45 ± 0.2ab	20.76 ± 1.13a
*Spartina*	No	2.75 ± 0.48a	7.35 ± 0.16a	7.26 ± 1.49a	0.15 ± 0.05a	1.83 ± 0.1ab	27.47 ± 1.38b
*Spartina*	Yes	3.15 ± 0.14a	7.62 ± 0.22a	10.43 ± 3.16a	0.44 ± 0.76a	1.75 ± 0.21b	26.8 ± 2.24b
ANOVA *P*-values							
Plant (P)		0.05	0.63	0.16	0.4	<0.01	<0.01
N (N enrichment)		0.24	0.4	0.23	0.24	0.7	1
P*N		0.52	0.05	0.04	0.71	0.29	0.4
**Plant type**	**N enrichment**	**P (mg kg**^**−1**^)	**Clay (%)**	**Silt (%)**	**C:N**	**N:P**	**C:P**
*Phragmites*	No	2.89 ± 0.13a	19.05 ± 5.11a	80.53 ± 4.9a	16.17 ± 3.66a	446 ± 92a	6962 ± 189a
*Phragmites*	Yes	3.08 ± 0.13a	22.43 ± 3.44a	77.03 ± 3.16a	14.45 ± 1.61a	472 ± 62a	6751 ± 404a
*Spartina*	No	2.04 ± 0.43b	20.66 ± 2.27a	79.3 ± 2.3a	15.07 ± 1.09a	926 ± 201b	13816 ± 2233b
*Spartina*	Yes	2.48 ± 0.41ab	22.4 ± 0.99a	77.59 ± 1a	15.5 ± 2.52a	726 ± 174b	11133 ± 2738b
ANOVA *P*-values							
P		<0.01	0.64	0.84	0.98	<0.01	<0.01
N		0.06	0.15	0.13	0.61	0.25	0.13
P*N		0.44	0.63	0.58	0.39	0.14	0.19

Treatments followed by the same letters are not significantly different.
